# Long noncoding RNA LINC02568 sequesters microRNA-874-3p to facilitate malignancy in breast cancer cells via cyclin E1 overexpression

**DOI:** 10.32604/or.2022.025172

**Published:** 2022-08-31

**Authors:** YI DONG, LIANBO ZHANG, XIN GUAN, TAO LIU, LIMIN ZHOU

**Affiliations:** 1 Department of Second Breast Surgery, Jilin Cancer Hospital, Jilin, 130012, China; 2 Department of Medical Insurance Guarantee Office, Jilin Cancer Hospital, Jilin, 130012, China; 3 Department of Breast Surgery, The First Hospital of Jilin University, Jilin, 130061, China

**Keywords:** Long noncoding RNA, ceRNA, Therapeutic intervention, microRNA

## Abstract

Increasing numbers of long noncoding RNAs (lncRNAs) are implicated in breast cancer oncogenicity. However, the contribution of LINC02568 toward breast cancer progression remains unclear and requires further investigation. Herein, we evaluated LINC02568 expression in breast cancer and clarified its effect on disease malignancy. We also investigated the mechanisms underlying the pro-oncogenic role of LINC02568. Consequently, LINC02568 was upregulated in breast cancer samples, with a notable association with worse overall survival. Functionally, depleted LINC02568 suppressed cell proliferation, colony formation, and metastasis, whereas LINC02568 overexpression exerted the opposite effects. Our mechanistic investigations suggested that LINC02568 was physically bound to and sequestered microRNA-874-3p (miR-874-3p). Furthermore, miR-874-3p mediated suppressive effects in breast cancer cells by targeting cyclin E1 (CCNE1). LINC02568 positively controlled CCNE1 expression by sequestering miR-874-3p. Rescue experiments revealed that increased miR-874-3p or decreased CCNE1 expression recovered cell growth and motility functions induced by LINC02568 in breast cancer cells. In conclusion, the tumor-promoting functions of LINC02568 in breast cancer cells were enhanced by sequestering miR-874-3p and consequently over-expressing CCNE1. Our data may facilitate the identification of novel therapeutic targets in clinical settings.

## Introduction

Globally, as a highly prevalent human malignancy, breast cancer is the leading cause of cancer-related mortality among women [1]. Each year, an estimated 2.1 million new breast cancer cases are cataloged, with approximately 0.63 million deaths [2]. Owing to tumor heterogeneity that is associated with high aggressiveness, breast cancer cells easily invade and extensively metastasize to distant organs, thereby decreasing the effectiveness of anticancer therapies and worsening clinical outcomes [3]. In recent decades, progress in diagnostic and management approaches has improved clinical efficacy in patients with breast cancer; however, advanced stage survival rates are far from satisfactory [4]. Therefore, the full elucidation of molecular events underlying breast cancer is warranted to identify effective targets for therapeutic intervention.

Despite the lack of protein-encoding capacity, long noncoding RNAs (lncRNAs) are promising anticancer therapeutic targets [5]. As a family of RNA transcripts comprising over 200 nucleotides, lncRNAs modulate many malignant processes, including cell proliferation, apoptosis, cell cycle, and key signaling pathways [6–8]. The aberrant expression of lncRNAs is significantly correlated with the genesis and development of human cancers [9], including breast cancer [10]. LncRNA dysregulation is critical during oncogenesis and breast cancer progression [11,12].

MicroRNAs (miRNAs) are a family of short, noncoding, single-stranded RNA transcripts [13]. They affect target mRNA expression by base-pairing with their 3′-untranslated regions to prevent translation and/or facilitate mRNA degradation [14]. Previously, the “competing endogenous RNA (ceRNA)” theory indicated that lncRNAs can control gene expression by decoying miRNAs; thus, lncRNAs serve as negative modulators of miRNAs and consequently positively regulate the targeted genes of miRNAs [15]. Therefore, lncRNAs and miRNAs are appropriate therapeutic targets for breast cancer.

lncRNA roles have received great interest in breast cancer research. Herein, we determined LINC02568 expression in breast cancer and clarified its roles. Additionally, we attempted to mechanistically determine if LINC02568 control in breast cancer progression was associated with the miR-874-3p/CCNE1 axis. Our findings may aid in the identification of attractive therapeutic targets for breast cancer management.

## Materials and Methods

### Patients and tissue specimens

This study was approved by the Ethics Committee of The First Hospital of Jilin University. Written informed consent was obtained from all participants. In total, 53 pairs of breast cancer tissues and matched para-cancer tissues were obtained. No participants previously underwent preoperative radiochemotherapy or immunotherapy.

### Quantitative reverse transcription–polymerase chain reaction (qRT–PCR)

Total RNA was isolated using TRIzol® (Invitrogen). RNA concentrations and purity were quantified using Nanodrop 2000 (Nanodrop; Thermo Fisher Scientific, Inc., Waltham, MA, USA,). To measure LINC02568 and CCNE1 levels, the PrimeScript® RT reagent kit (TaKaRa, Dalian, China) was used to synthesize cDNA and PCR was performed using SYBR® Premix Ex TaqTM II (TaKaRa). LINC02568 and CCNE1 levels were normalized to glyceraldehyde 3-phosphate dehydrogenase (GAPDH). To detect miR-874-3p expression, cDNA synthesis was performed using the Mir-X miRNA first-strand synthesis kit (TaKaRa). qPCR was performed using the Mir-X miRNA qRT-PCR TB Green® kit (TaKaRa). U6 was used as a reference gene for miR-874-3p normalization. The 2^-ΔΔCq^ method was used for data analysis.

### Cell lines

The breast cancer epithelial cell line MCF-10A (American Type Culture Collection [ATCC], Manassas, VA, USA) was cultured in 100 ng/mL cholera toxin-supplemented Mammary Epithelial Cell Growth Medium BulletKit^TM^ (Lonza/Clonetics Corporation, Walkersville, MD, USA). The breast cancer cell lines MCF-7 and MDA-MB-468 were obtained from the Chinese Academy of Medical Sciences (Shanghai, China). MCF-7 cells were maintained in minimum essential medium supplemented with 10% fetal bovine serum ([FBS]; Gibco). The breast cancer cell lines MDA-MB-231 and SK-BR-3 (ATCC) were cultured in L-15 and McCoy’s 5A medium (Gibco), both of which were supplemented with 10% FBS. The culture conditions for MDA-MB-468 cells were the same as for MDA-MB-231 cells. All cells were cultured at 37°C in saturated humidity under 5% CO_2_ atmosphere.

### Transfection

Two small interfering RNAs (siRNAs) targeting LINC02568 (si-LINC02568) and CCNE1 (si-CCNE1) and a negative control (NC) siRNA (si-NC) were synthesized by GenePharma (Shanghai, China). The LINC02568 overexpression plasmid pcDNA3.1-LINC02568 (pc-LINC02568) and CCNE1 overexpression plasmid pcDNA3.1-CCNE1 (pc-CCNE1) were also provided by GenePharma. RiboBio (Guangzhou, China) provided the miR-874-3p mimic, NC mimic, miR-874-3p inhibitor, and NC inhibitor. Cells were transfected with the abovementioned molecular products using Lipofectamine® 2000 (Invitrogen).

### Cell counting kit-8 (CCK-8) assay

Transfected cells were harvested at 24 h post-transfection and used to prepare cell suspensions. Next, 100 μL cell suspensions were seeded into a 96-well plate at a density of 2 × 10^3^ cells/well and cultured overnight. To determine cell proliferation, 10 μL of CCK-8 solution (Dojindo, Kumamoto, Japan) was added to the wells for 2 h at 0, 1, 2, and 3 days after transfection. Absorbance at 450 nm was measured on an iMark microplate reader (Bio-Rad, Munchen, Germany).

### Colony formation assay

To analyze colony formation capacity, cell suspension concentrations were adjusted to 500 cells/mL. A total of 2 mL cell suspension was added into 6-well plates and cultured for 14 days at 37°C under 5% CO_2_. Finally, colonies were fixed with 4% formaldehyde and dyed using 0.1% crystal violet. Colonies were counted under a light microscope (Olympus).

### Transwell migration and invasion assays

To detect migratory capacity, single-cell suspensions were generated in serum-free medium. To the upper chambers of a transwell plate (BD Biosciences), a 600 μL cell suspension containing 5 × 10^4^ cells was added. Lower chambers contained 10% FBS-supplemented culture medium. Transwell chambers were incubated for 24 h. After incubation, nonmigrated cells were wiped away using a cotton swab, whereas migrated cells were fixed with 4% formaldehyde and dyed using 0.1% crystal violet. To measure cell invasion, the upper filter was pre-coated in Matrigel (BD Biosciences). Subsequent steps were the same as those of the migration assay. Cells that passed through membranes were photographed under a light microscope (magnification value: x200).

### Xenograft tumor model

Short hairpin RNA (shRNA) against LINC02568 (sh-LINC02568) and negative controls (sh-NCs) were purchased from GenePharma, cloned into the pLKO.1 vector (Addgene Inc., Watertown, MA, USA), and transfected into 293T cells (Chinese Academy of Medical Sciences) together with the psPAX2 and pMD2.G lentiviral plasmids (Addgene Inc.). After culturing for 2 days at 37°C in saturated humidity under 5% CO_2_, the plasmids harboring either sh-LINC02568 or sh-NC were harvested and transfected into MCF-7 cells. Finally, puromycin was added to select stably transfected cells.

All animal experiments were approved by the First Hospital of Jilin University. Approximately 1 × 10^6^ cells stably transfected with sh-LINC02568 or sh-NC were subcutaneously inoculated into BALB/c female nude mice. The status of newly formed tumors was observed every 4 days. Tumor width and length were recorded to determine tumor volume. At 31 days after tumor cell injection, mice were humanely euthanized and subcutaneous tumors were removed and photographed.

### Nuclear–cytoplasmic fractionation

The experiment was performed using the Cytoplasmic and Nuclear RNA Purification Kit (Norgen, Thorold, ON, Canada) according to manufacturer’s instructions. Cytoplasmic and nuclear fractions were obtained and subjected to RNA extraction using TRIzol (Invitrogen). Next, the relative distribution of LINC02568 in breast cancer cells was determined using qRT–PCR.

### RNA immunoprecipitation (RIP) assay

With the aim to verify the interaction among LINC02568, miR-874-3p and CCNE1, RIP assay was conducted employing a Magna RIP RNA Binding Protein Immunoprecipitation kit (Millipore, Darmstadt, Germany). Breast cancer cells were cultivated with RIP-lysis buffer, and the whole breast cancer cell lysates were acquired. The cell lysates were then probed with magnetic beads that were conjugated with Ago2) antibody or IgG. The incubation process was implemented at 4°C, and lasted all night. After that, the magnetic beads were collected, and washed successively using wash buffers. The coprecipitated RNA was isolated, and analyzed with qRT-PCR.

### Bioinformatics tools

The putative targets of LINC02568 were predicted utilizing starBase 3.0 (https://starbase.sysu.edu.cn/) and miRDB (http://mirdb.org/). TargetScan (http://www.targetscan.org/) and miRDB were adopted for predicting the binding between miR-874-3p and CCNE1.

### Luciferase reporter assay

LINC02568 and CCNE1 fragments harboring wild-type (WT) miR-874-3p binding sequences were amplified by GenePharma. After being inserted into the pmirGLO vector, the luciferase reporter vectors, namely WT-LINC02568 and WT-CCNE1, were constructed. Meanwhile, the mutant (MUT) luciferase reporter vectors, namely MUT-LINC02568 and MUT-CCNE1, were also synthesized. For reporter assay, miR-874-3p mimic or NC mimic together with reporter plasmids were transfected into breast cancer cells. Forty-eight hours later, luciferase activity was detected by means of a Dual-Luciferase Reporter Assay System (Promega, Madison, WI, USA).

### Western blotting

Total protein extraction was performed using RIPA lysis buffer supplemented with phenylmethylsulfonyl fluoride and protease inhibitors (Beyotime). After quantifying proteins using a bicinchoninic acid assay kit (Beyotime), equivalent quantities were separated using 10% sodium dodecyl sulfate–polyacrylamide gel electrophoresis and electrophoretically transferred onto polyvinylidene fluoride membranes. After blocking in 5% milk, membranes were incubated with primary antibodies targeting CCNE1 (ab33911) or GAPDH (ab128915; Abcam, Cambridge, UK). After overnight incubation at 37°C, membranes were washed and incubated with a secondary antibody for 1 h. Protein signals were visualized using BeyoECL Plus Western Blotting Detection Reagent (Beyotime). ImageJ software (National Institutes Health, MD, USA) was used to determine protein gray density signals.

### Statistical analysis

All experiments were repeated at least three times, and data are presented as the mean ± standard deviation. Two-group comparisons were performed using Student’s *t*-test. A one-way analysis of variance followed by a Bonferroni *post hoc* test was used to perform multigroup comparisons. The Kaplan–Meier method was used to generate survival curves, which were analyzed using log-rank test. Data analyses were performed using SPSS 22.0 software (IBM Corp., Armonk, NY, USA). A *P* value of <0.05 was considered statistically significant.

## Results

### LINC02568 is overexpressed in breast cancer

**Figure 1:**
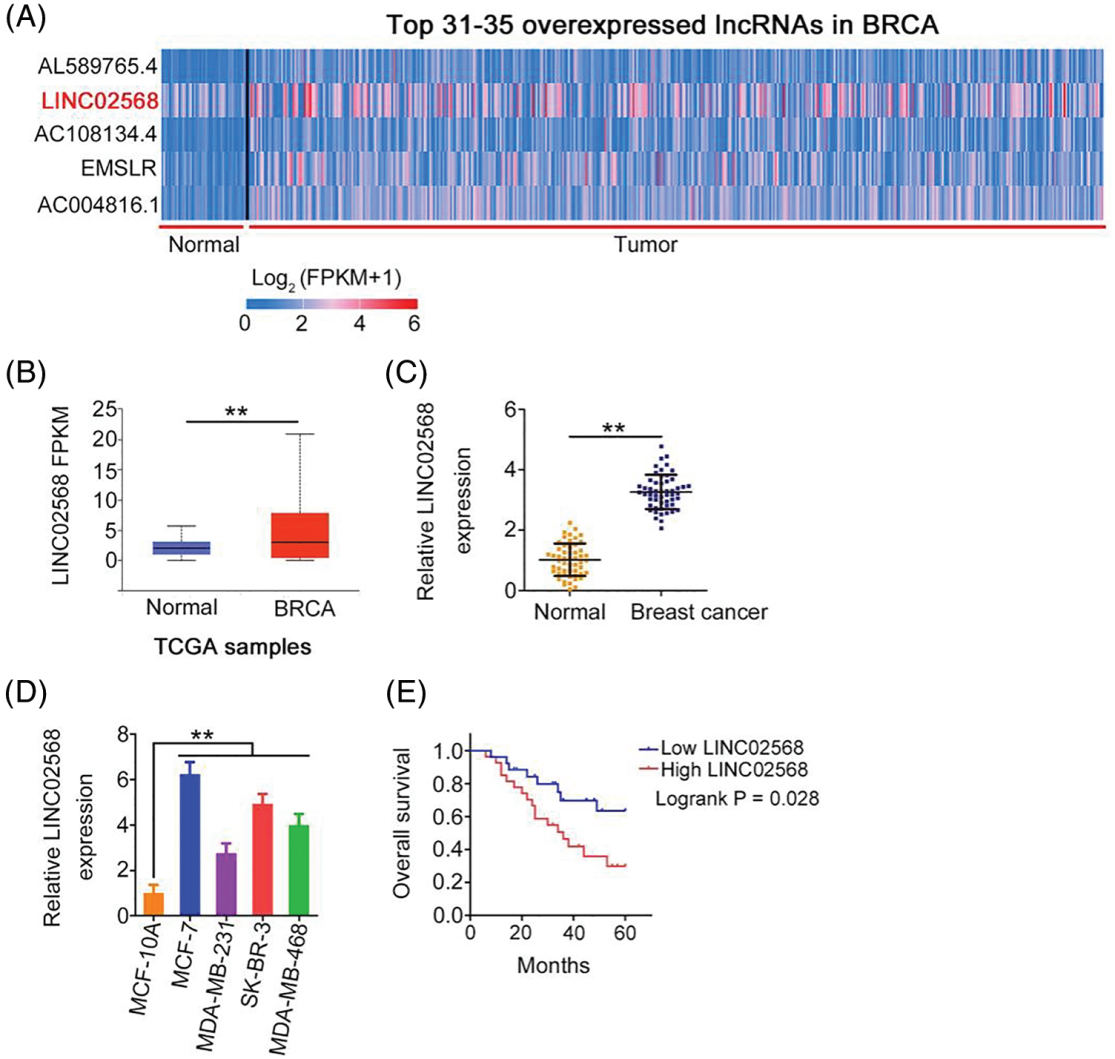
LINC02568 was upregulated in breast cancer. (A) LINC02568 rans the 32^th^ overexpressed lncRNA in BRCA. (B) LINC02568 in BRCA tissue samples from TCGA dataset. (C) LINC02568 expression in breast cancer tissues from our cohort. (D) LINC02568 level in breast cancer cell lines. (E) Overall survival curve manifested the prognosis of breast cancer patients with high or low LINC02568 level. **p < 0.001 (n = 3).

Using The Cancer Genome Atlas (TCGA) database, we analyzed differentially expressed lncRNAs in invasive breast carcinoma (BRCA). LINC02568 was the 32^nd^ most overexpressed lncRNA in BRCA (Fig. 1A); therefore, we selected it as a candidate gene in our breast cancer analysis. As shown in Fig. 1B, significant LINC02568 overexpression was identified in breast cancer tissues. We also measured LINC02568 expression in breast cancer tissues from our cohort; compared with matched para-cancer tissues, LINC02568 was overexpressed in breast cancer tissues (Fig. 1C). As shown in Fig. 1D, LINC02568 was upregulated in all selected breast cancer cell lines. Furthermore, high LINC02568 levels were associated with worse prognosis in patients with breast cancer (Fig. 1E).

### LINC02568 depletion suppresses proliferation, colony formation, and motility of breast cancer cells

**Figure 2:**
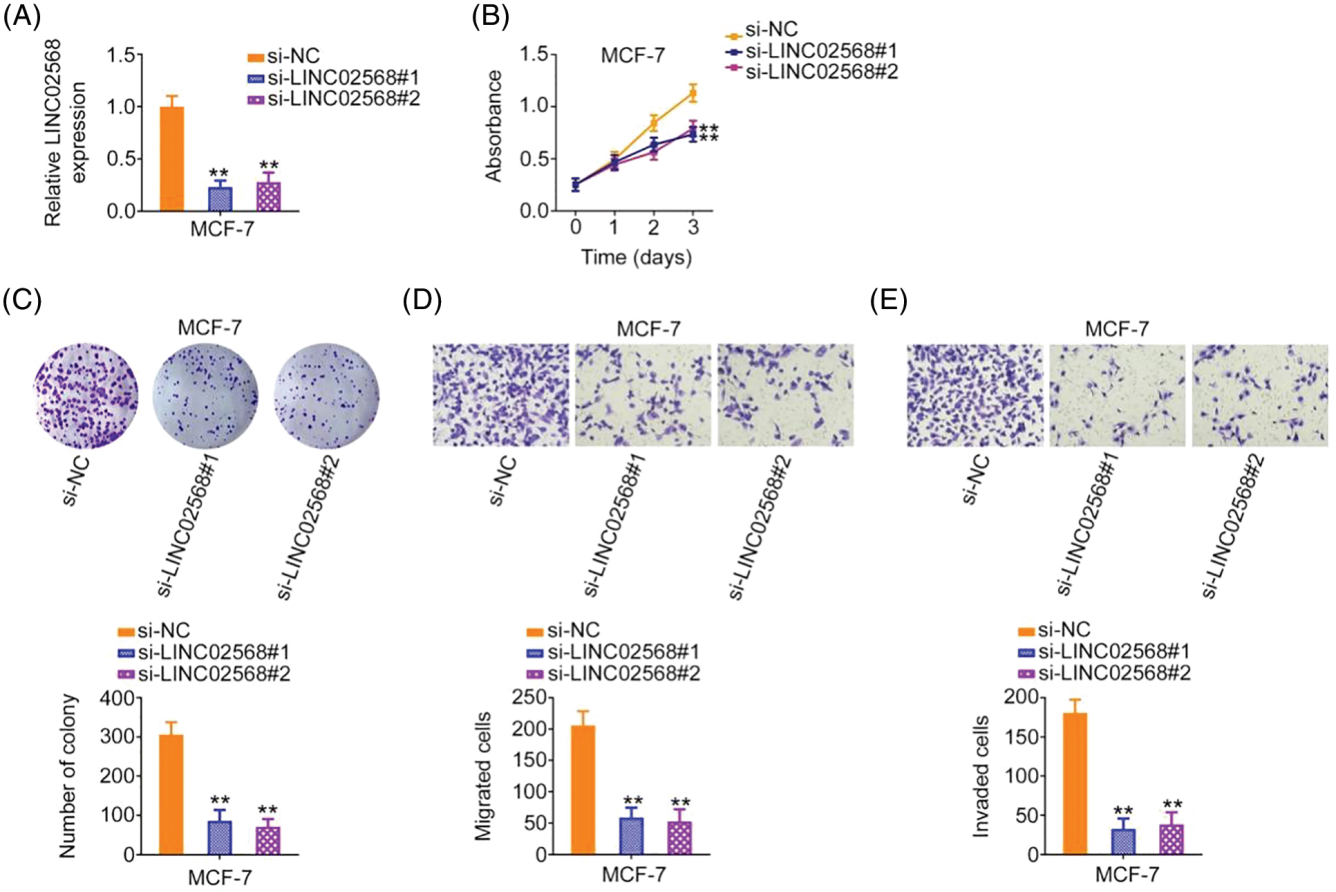
LINC02568 deficiency represses the growth and motility of MCF-7 cells. (A) Transfection efficiency of si-LINC02568 in MCF-7 cells. (B, C) The proliferation of LINC02568-silenced MCF-7 cells. (D, E) The motility of MCF-7 cells transfected with si-LINC02568. 100× magnification. **p < 0.001 (n = 3).

We investigated whether ablating LINC02568 would affect breast cancer cell aggressiveness. Of the four breast cancer cell lines, the highest LINC02568 levels were detected in MCF-7 cells; therefore, these cells were chosen for loss-of-function studies. si-LINC02568 #1 and si-LINC02568 #2 were used to silence LINC02568 in cells (Fig. 2A). Cell proliferation capacity was notably decreased after LINC02568 silencing (Fig. 2B). Additionally, colony formation was suppressed by elevating LINC02568 expression (Fig. 2C). Furthermore, as shown in Figs. 2D and 2E, downregulated LINC02568 repressed MCF-7 cell migratory and invasive abilities.

### LINC02568 upregulation stimulates breast cancer cell proliferation, colony formation, and motility

**Figure 3:**
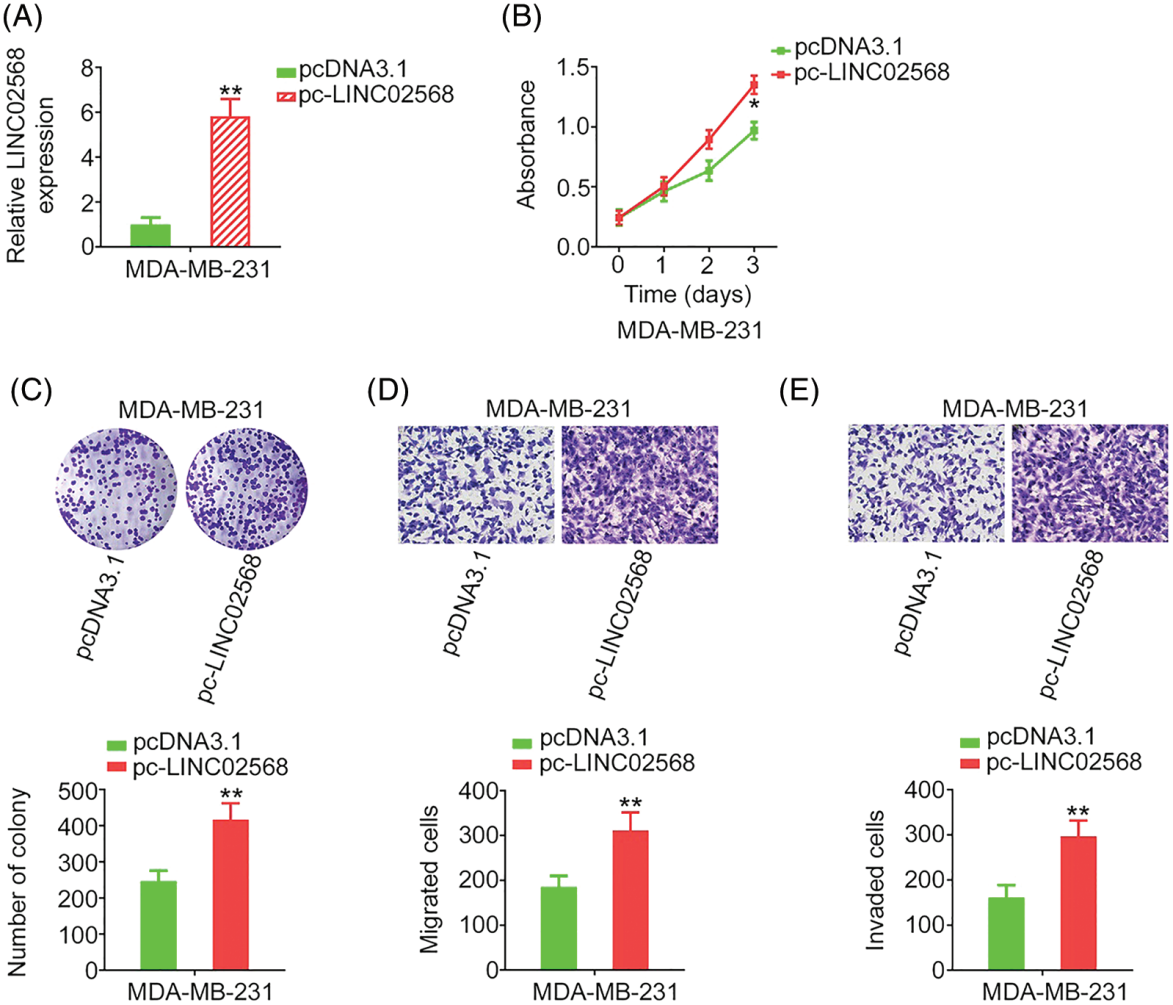
LINC02568 overexpression facilitated the growth and motility of MDA-MB-231 cells. (A) Transfection efficiency of pc-LINC02568 in MDA-MB-231 cells. (B, C) The proliferation of LINC02568-overexpressed MDA-MB-231 cells. (D, E) The motility of LINC02568-overexpressed MDA-MB-231 cells. 100× magnification. *p < 0.01 and **p < 0.001 (n = 3).

LINC02568 gain-of-function studies were conducted using the LINC02568 overexpression plasmid pc-LINC02568. MDA-MB-231 cells had the lowest LINC02568 levels; therefore, these cells were used for gain-of-function studies. LINC02568 levels were remarkably overexpressed by pc-LINC02568 in MDA-MB-231 cells (Fig. 3A). Upregulation of LINC02568 considerably stimulated the proliferative and clonogenic abilities of MDA-MB-231 cells (Figs. 3B and 3C). Moreover, we assessed the regulatory effect of pc-LINC02568 on cell motility; in the pc-LINC02568 group, enhanced cell motility was observed (Figs. 3D and 3E). Thus, LINC02568 functioned as a tumor promoter during breast cancer progression.

### LINC02568 functions as an miR-874-3p sponge

**Figure 4:**
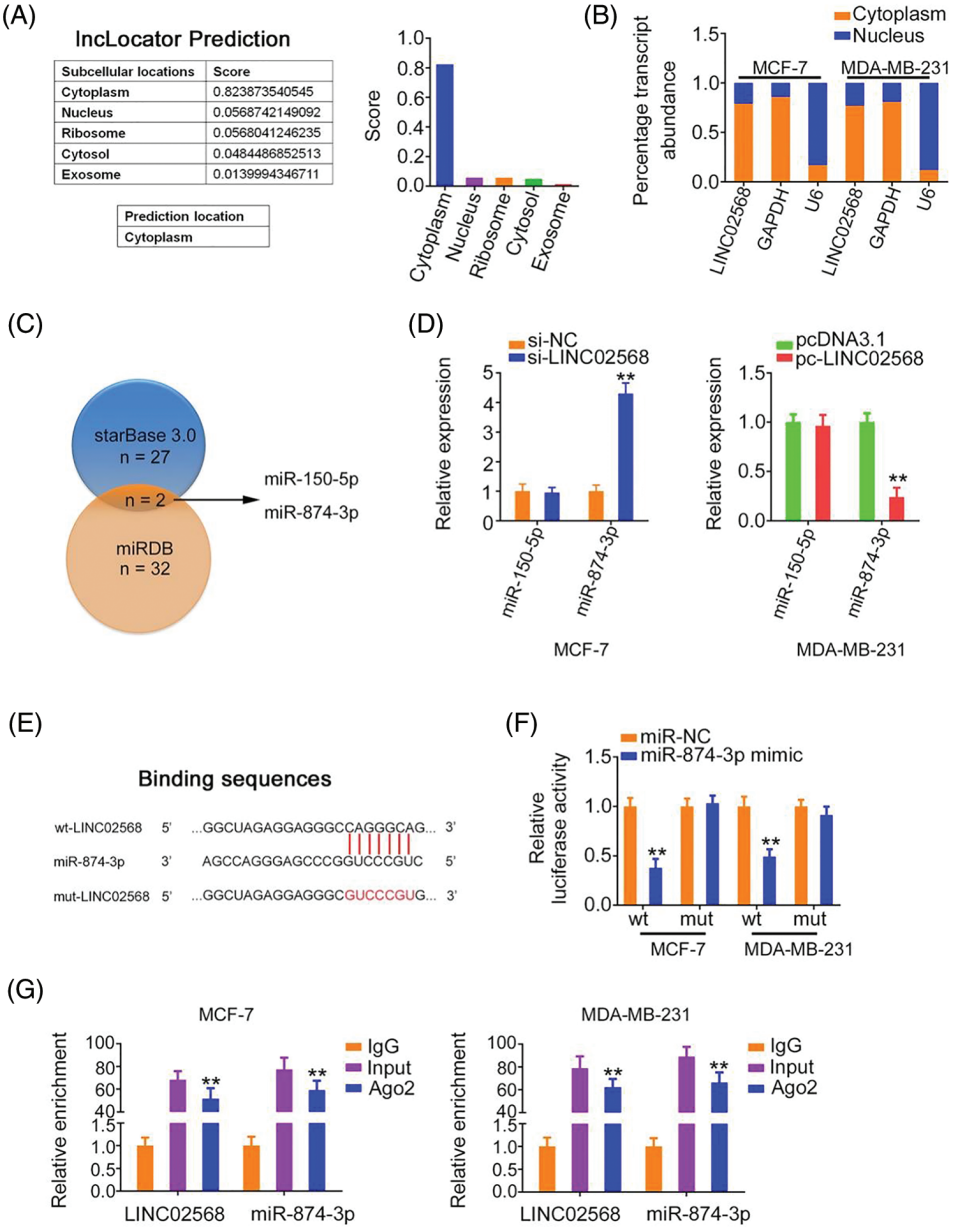
LINC02568 sponged miR-874-3p in breast cancer. (A) Localization of LINC02568 predicted by lncLocator Prediction. (B) The localization of LINC02568 in breast cancer cells was determined utilizing nuclear-cytoplasmic fractionation. (C) The overlapping miRNAs targeted by starBase 3.0 and miRDB. (D) MiR-150-5p and miR-874-3p levels in LINC02568-silenced MCF-7 cells. Also, their expression in MDA-MB-231 cells after pc-LINC02568 transfection was examined. (E) The predicted binding sequences between LINC02568 and miR-874-3p. (F) Luciferase reporter assay of the binding between LINC02568 and miR-874-3p. (G) RIP was performed applying an Ago2 antibody, and the enrichment of LINC02568 and miR-874-3p was evaluated. *p < 0.001 (n = 3).

To characterize the molecular events underlying the effect of LINC02568 during tumorigenesis, we determined its localization in breast cancer cells. Using lncLocator, we predicted the abundant subcellular location of LINC02568 in the cytoplasm (Fig. 4A). Our nuclear–cytoplasmic fractionation experiment further verified that most LINC02568s were indeed distributed in the cytoplasm of breast cancer cells (Fig. 4B). Therefore, LINC02568 may play pro-oncogenic roles as a ceRNA or molecular sponge to sequester certain miRNAs.

We used bioinformatics algorithms to search for miRNAs that may have been sequestered by LINC02568. Two overlapping miRNAs (miR-150-5p and miR-874-3p) were identified from the miRDB and starBase 3.0 databases (Fig. 4C). We detected the expression of both in breast cancer cells following LINC02568 alterations. LINC02568 downregulation resulted in miR-874-3p upregulation, whereas ectopic LINC02568 expression decreased miR-874-3p expression (Fig. 4D). The miR-874-3p region targeted by LINC02568 is shown in Fig. 4E. Luciferase activity was evidently downregulated in cells cotransfected with the WT-LINC02568 and miR-874-3p mimic, whereas MUT-LINC02568 showed no remarkable change in luciferase activity in response to miR-874-3p mimic cotransfection (Fig. 4F). Moreover, as anticipated by the radioimmunoprecipitation experiment, LINC02568 and miR-874-3p enrichment was verified in Ago2 precipitates, which implied their binding interactions in breast cancer cells (Fig. 4G). Taken together, LINC02568 functioned as an miR-874-3p sponge.

### MiR-874-3p suppresses breast cancer progression and directly targets CCNE1

**Figure 5:**
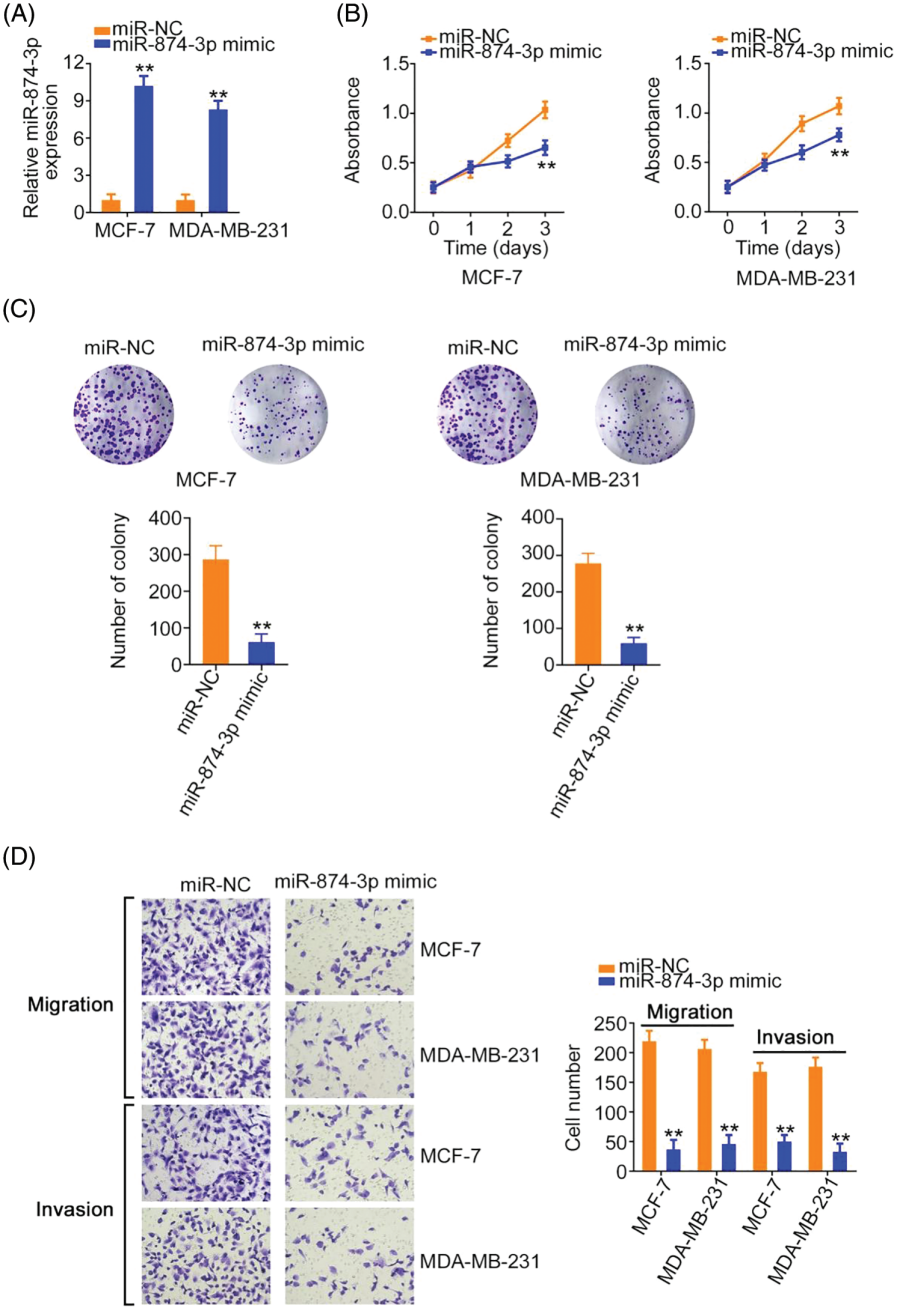
miR-874-3p was characterized as a tumor-suppressing miRNA. (A) The efficiency of miR-874-3p mimic transfection. (B, C) The proliferation of miR-874-3p-overexpressed cells. (D) The motility of miR-874-3p-overexpressed cells. 100× magnification. **p < 0.001 (n = 3).

MiR-874-3p levels were increased in breast cancer cells after miR-874-3p mimic transfection (Fig. 5A). Cell proliferative and clonogenic abilities were decreased by the miR-874-3p mimic (Figs. 5B and 5C). Furthermore, the effect of miR-874-3p on cell motility was explored using transwell migration and invasion assays. As shown in Fig. 5D, compared with the NC mimic, cell motility was eliminated by miR-874-3p overexpression.

**Figure 6:**
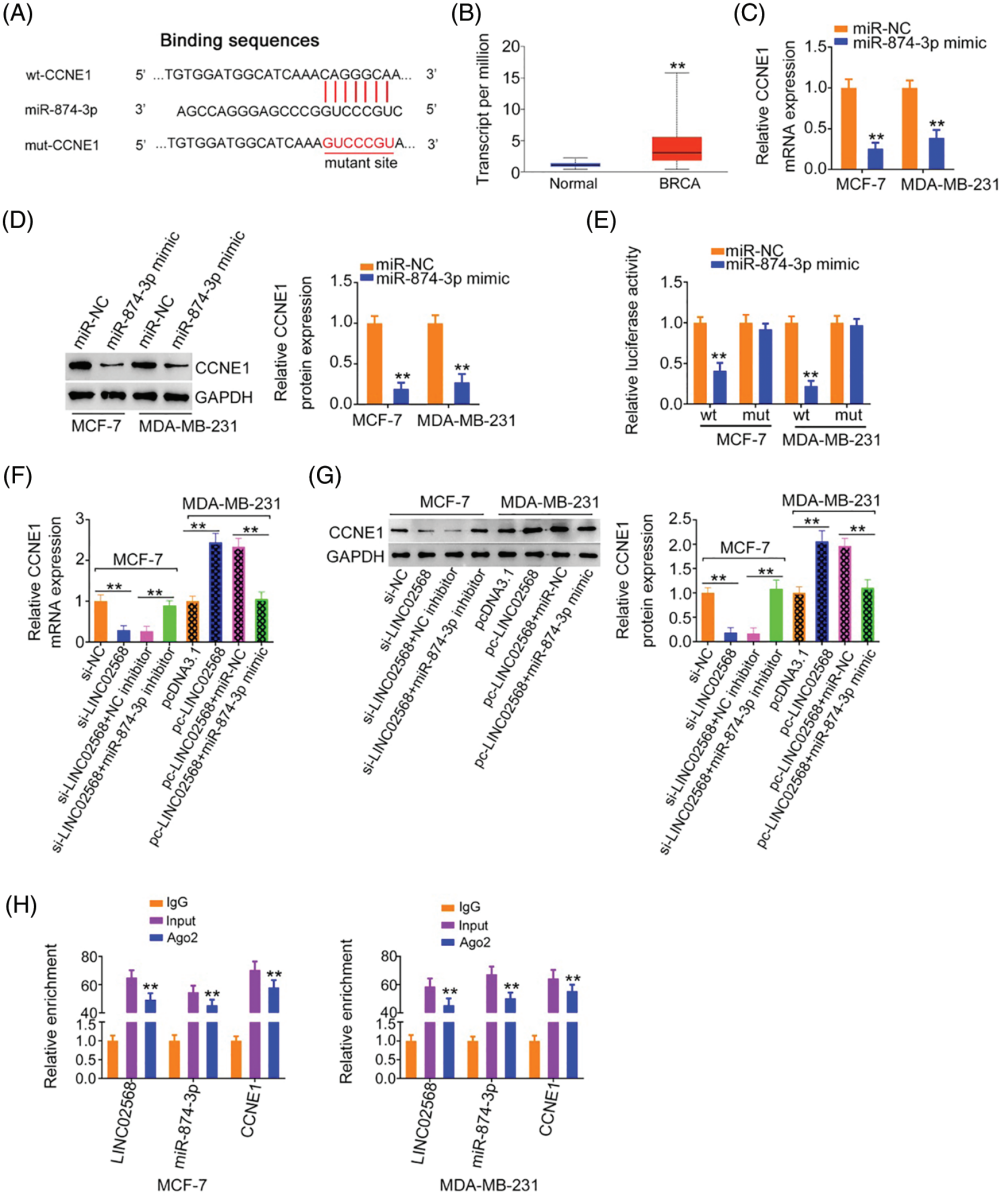
CCNE1 was modulated by LINC02568/miR-874-3p axis. (A) The binding site between CCNE1 and miR-874-3p. (B) CCNE1 expression in BRCA tissue samples from TCGA dataset. (C, D) CCNE1 levels in breast cancer cells after miR-874-3p upregulation. (E) Luciferase reporter assay of the interaction between CCNE1 and miR-874-3p. (F, G) MCF-7 cells were transfected with si-NC, si-LINC02568, si-LINC02568+NC inhibitor, or si-LINC02568+miR-874-3p inhibitor. MDA-MB-231 cells were transfected with pcDNA3.1, pcLINC02568, pc-LINC02568+miR-NC, or pc-LINC02568+miR-874-3p mimic. After transfection, CCNE1 mRNA and protein expression was measured. (H) RIP was performed applying an Ago2 antibody, and the enrichment of LINC02568, miR-874-3p and CCNE1 was detected. **p < 0.001 (n = 3).

Using a bioinformatics algorithm, CCNE1 was predicted as a candidate of miR-874-3p (Fig. 6A). Using TCGA database, we found that CCNE1 was obviously overexpressed in BRCA (Fig. 6B). Additionally, CCNE1 exerts important regulatory actions in breast cancer progression [16–20]. Thus, CCNE1 was selected for further verification. CCNE1 expression was detected after miR-874-3p upregulation. Overexpressed miR-874-3p induced a notable decrease in CCNE1 levels in cells (Figs. 6C and 6D). Additionally, luciferase reporter assays showed that the miR-874-3p mimic remarkably reduced WT-CCNE1-induced luciferase activity; however, the mutation of the predicted binding sequences completely reversed these suppressive effects (Fig. 6E). Thus, we confirmed direct binding between miR-874-3p and CCNE1 in breast cancer cells.

### LINC02568 negatively regulates CCNE1 expression in breast cancer cells by decoying miR-874-3p

According to the abovementioned findings, we determined that LINC02568 sponged miR-874-3p, with the latter directly targeting CCNE1. Next, we examined if LINC02568 affected CCNE1 expression and possible underlying mechanisms. LINC02568 knockdown decreased CCNE1 expression in MCF-7 cells; however, treatment with the miR-874-3p inhibitor reversed the effects of si-LINC02568 on CCNE1 (Figs. 6F and 6G) levels. In addition, LINC02568 upregulation increased CCNE1 levels in MDA-MB-231 cells, whereas cotransfection with the miR-874-3p mimic mitigated CCNE1 overexpression induced by pc-LINC02568 (Figs. 6F and 6G). Furthermore, LINC02568, miR-874-3p, and CCNE1 enrichment was verified in Ago2 precipitates (Fig. 6H), which suggested direct interactions between the three molecules. Thus, LINC02568 sponged miR-874-3p and consequently increased CCNE1 levels.

### LINC02568 worsens breast cancer oncogenicity by targeting miR-874-3p/CCNE1

**Figure 7:**
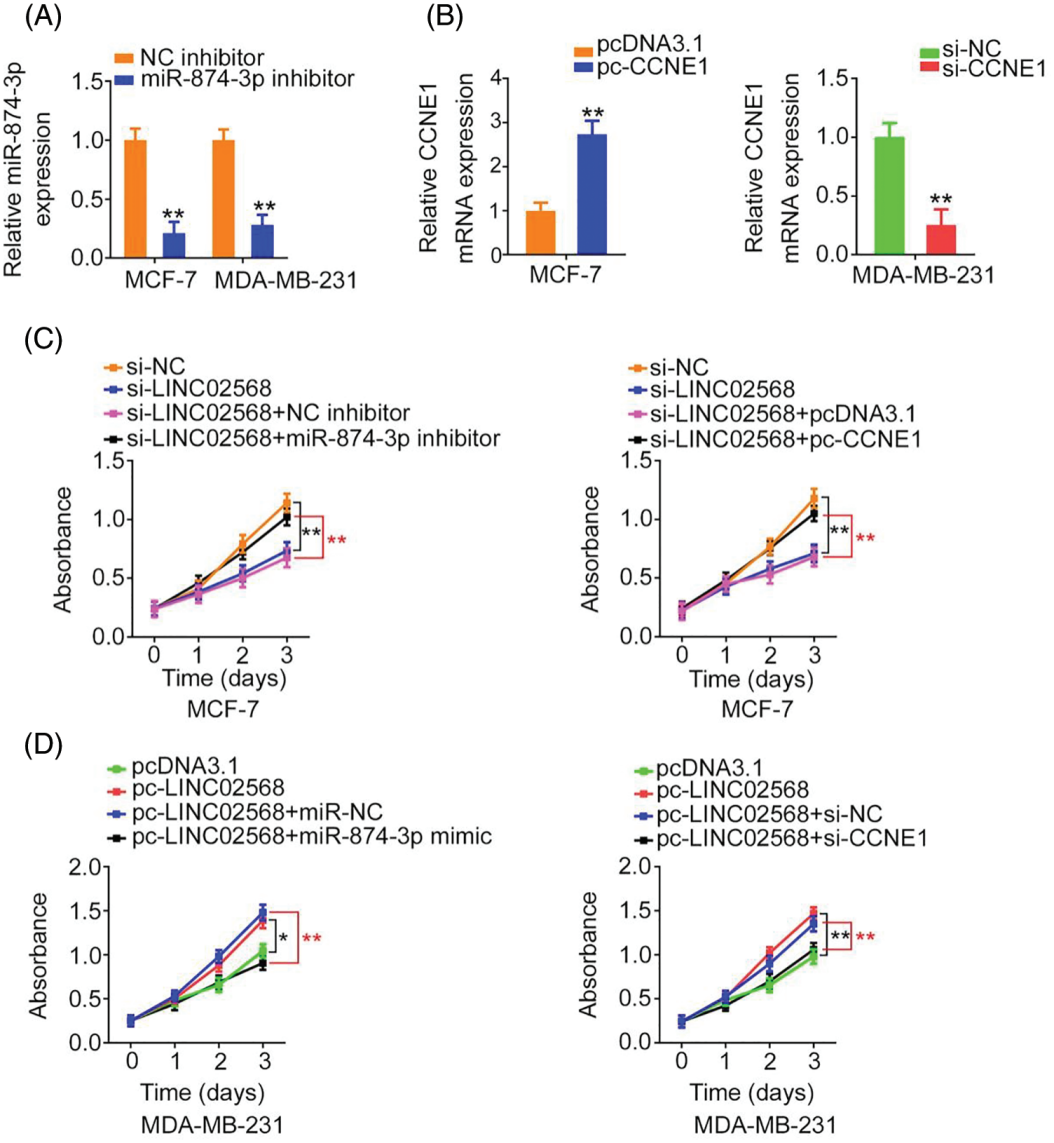
LINC02568 regulated the growth of breast cancer cells via targeting CCNE1/miR-874-3p. (A) The transfection efficiency of miR-874-3p inhibitor. (B) The efficiency of pc-CCNE1 in MCF-7 cells, and si-CCNE1 in MDA-MB-231 cells. (C) MCF-7 cells were treated with si-NC, si-LINC02568, si-LINC02568+NC inhibitor, si-LINC02568+miR-874-3p inhibitor, si-LINC02568+pcDNA3.1, si-LINC02568+pc-CCNE1. After transfection, cell proliferation in each group was determined. (D) MDA-MB-231 cells were transfected with pcDNA3.1, pc-LINC02568, pc-LINC02568+miR-NC, pc-LINC02568+miR-874-3p mimic, pc-LINC02568+si-NC, or pc-LINC02568+si-CCNE1. After transfection, cell proliferation in each group was determined. *p < 0.01 and **p < 0.001 (n = 3).

**Figure 8:**
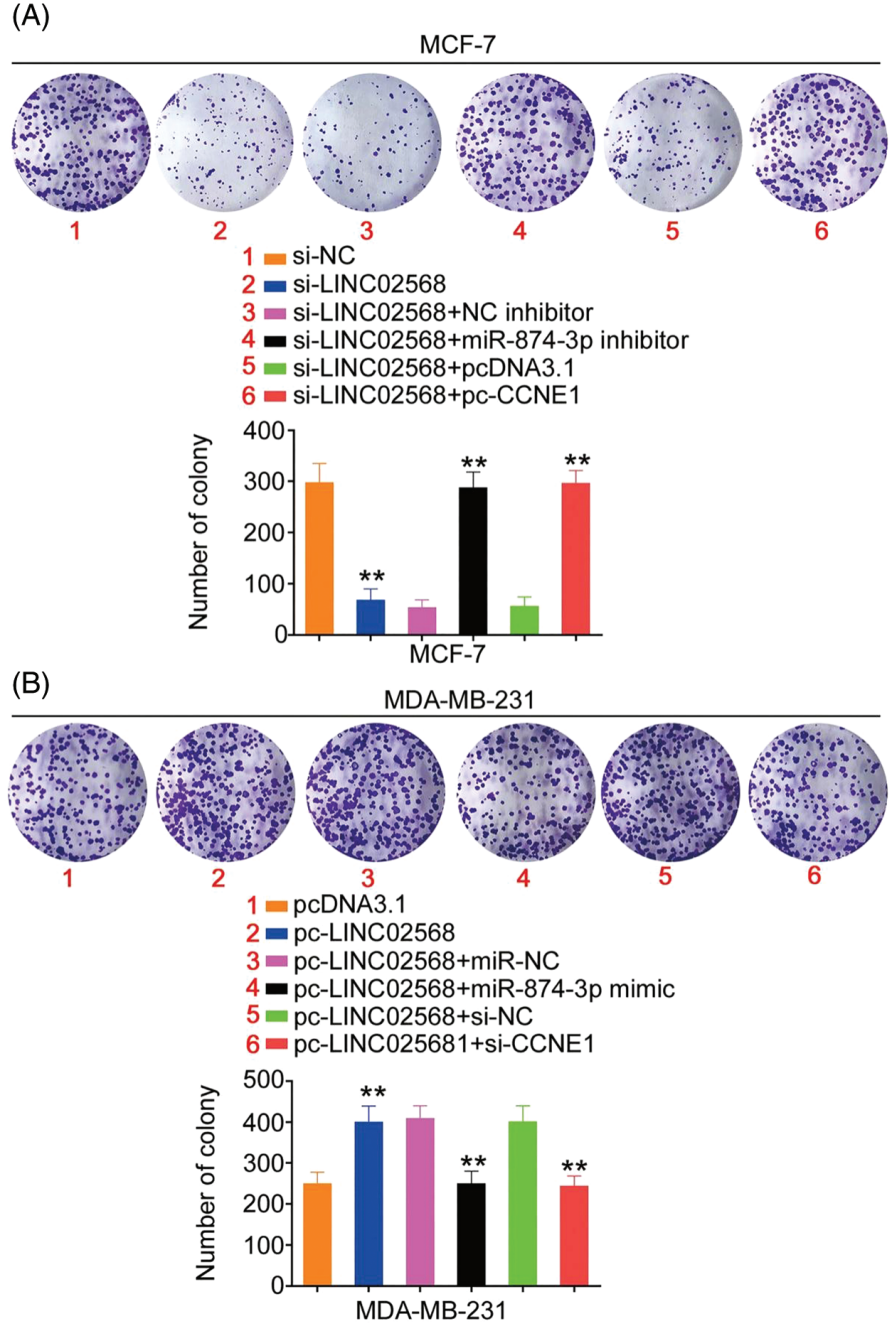
CCNE1/miR-874-3p axis was responsible for the regulatory effect of LINC02568 on cell colony formation. (A) MCF-7 cells were treated with si-NC, si-LINC02568, si-LINC02568+NC inhibitor, si-LINC02568+miR-874-3p inhibitor, si-LINC02568+pcDNA3.1, si-LINC02568+pc-CCNE1. Colony formation assay was implemented to detect cell colony formation. (B) MDA-MB-231 cells were transfected with pcDNA3.1, pc-LINC02568, pc-LINC02568+miR-NC, pc-LINC02568+miR-874-3p mimic, pc-LINC02568+si-NC, or pc-LINC02568+si-CCNE1.Colony formation assay was implemented to detect cell colony formation. **p < 0.001 (n = 3).

**Figure 9:**
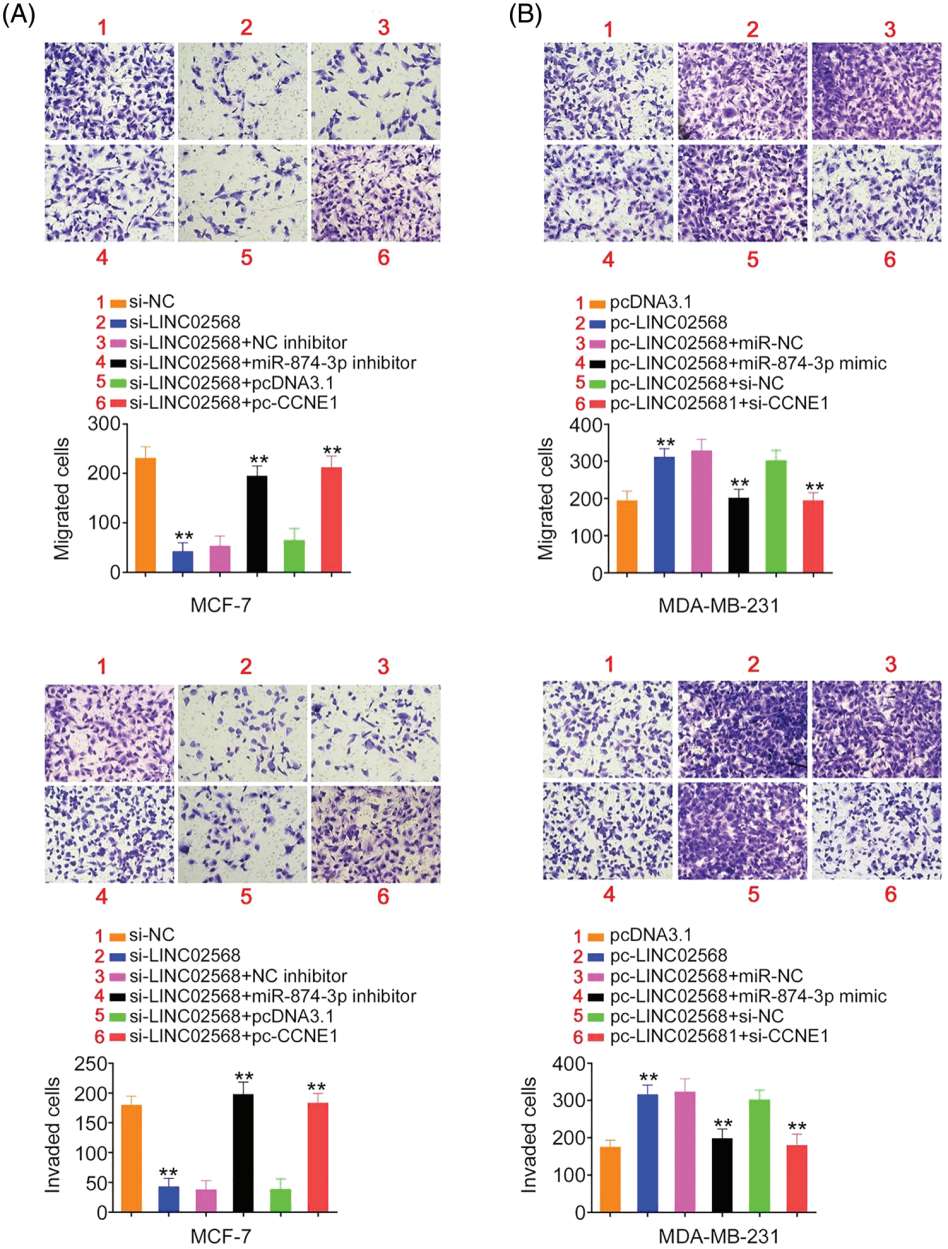
LINC02568 promoted breast cancer cell metastasis by controlling CCNE1/miR-874-3p axis. (A) MCF-7 cells were treated with si-NC, si-LINC02568, si-LINC02568+NC inhibitor, si-LINC02568+miR-874-3p inhibitor, si-LINC02568+pcDNA3.1, si-LINC02568+pc-CCNE1. The motility in each group was analysed. (B) MDA-MB-231 cells were transfected with pcDNA3.1, pc-LINC02568, pc-LINC02568+miR-NC, pc-LINC02568+miR-874-3p mimic, pc-LINC02568+si-NC, or pc-LINC02568+si-CCNE1. The motility in each group was analysed. **p < 0.001 (n = 3).

Rescue studies were used to validate the role of the miR-874-3p/CCNE1 axis on the pro-oncogenic roles of LINC02568. Transfection efficiencies of miR-874-3p inhibitor, si-CCNE1, or pc-CCNE1 were measured (Figs. 7A and 7B). MCF-7 cells were transfected with si-LINC02568 and the miR-874-3p inhibitor or pc-CCNE1, whereas MDA-MB-231 cells were transfected with the miR-874-3p mimic or si-CCNE1 and pc-LINC02568. The repressive effects of si-LINC02568 on MCF-7 cell proliferation (Fig. 7C) were reversed by downregulating miR-874-3p or overexpressing CCNE1. The proliferative (Fig. 7D) ability of MDA-MB-231 cells were promoted by pc-LINC02568, and these effects were reversed by the miR-874-3p mimic or si-CCNE1. In addition, the colony formation of MCF-7 cells with si-LINC02568 transfection was hindered but restored by miR-874-3p inhibitor or pc-CCNE1 cotransfection (Fig. 8A); meanwhile, the promoted colony formation of pc-LINC02568 transfected MDA-MB-231 cells was recovered after cotransfection of miR-874-3p mimic or si-CCNE1 (Fig. 8B). Furthermore, the motility of si-LINC02568-transfected MCF-7 cells was impaired but restored by cotransfection with the miR-874-3p inhibitor or pc-CCNE1 (Fig. 9A). Moreover, LINC02568 upregulation expedited MDA-MB-231 cell motility, whereas increasing miR-874-3p or deceasing CCNE1 levels counteracted these effects (Fig. 9B). Thus, LINC02568 aggravated breast cancer malignancy by modulating miR-874-3p/CCNE1.

### LINC02568 ablation impairs xenograft tumor growth in vivo

**Figure 10:**
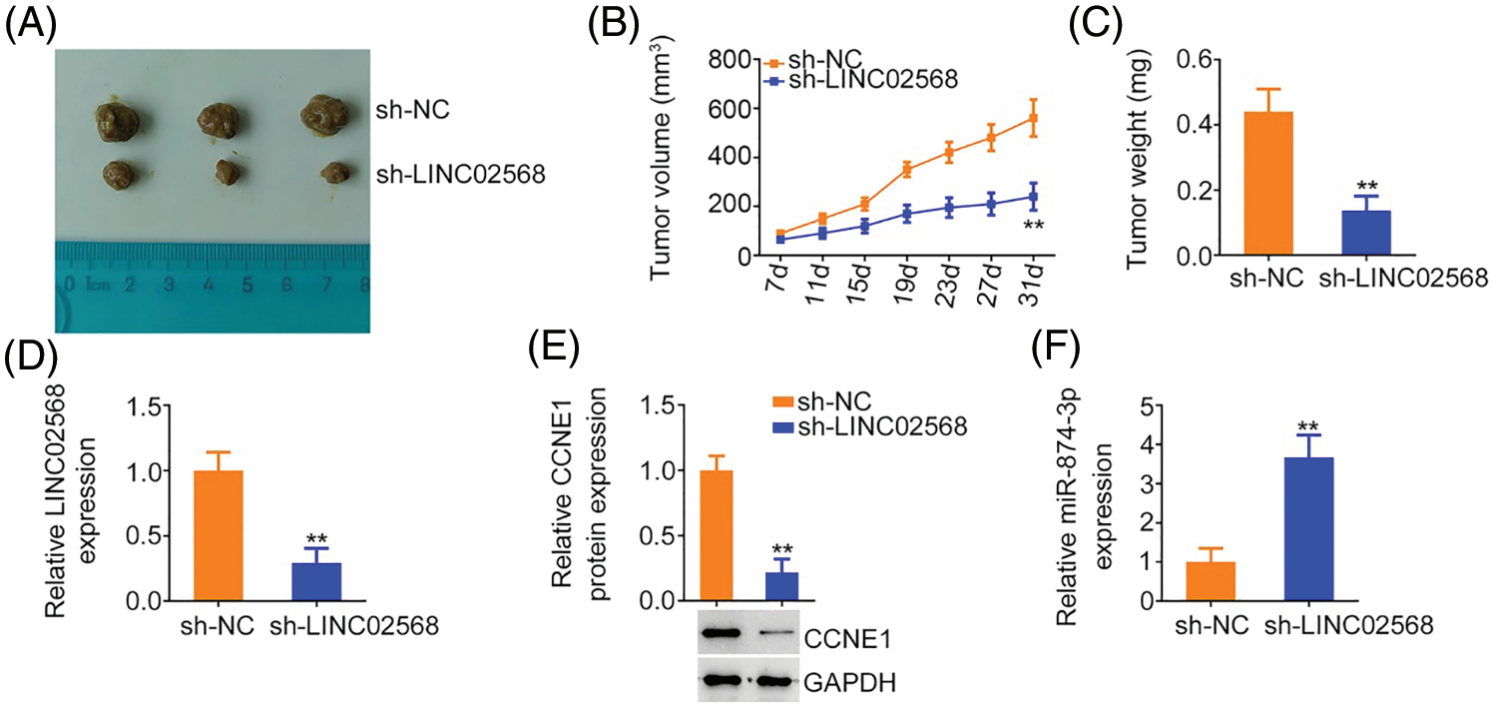
LINC02568 deficiency hampers tumor growth *in vivo*. (A) Xenografts from nude mice injected with sh-NC and sh-LINC02568. (B) Tumor volumes of xenografts in groups sh-NC and sh-LINC02568. (C) The wright of xenografts. (D–F) LINC02568, CCNE1 and miR-874-3p levels in xenografts. **p < 0.001 (n = 3).

MCF-7 cells stably expressing sh-LINC02568 or sh-NC were inoculated into nude mice to explore whether LINC02568 functioned as a tumor promoter *in vivo*. As indicated by the tumor growth curves in Figs. 10A and 10B, LINC02568 depletion impaired xenograft tumor growth *in vivo*. Subcutaneous xenograft tumors were also weighted; tumor weights in mice injected with sh-LINC02568 were decreased in contrast to those in mice injected with sh-NC (Fig. 10C). Furthermore, LINC02568 (Fig. 10D) and CCNE1 (Fig. 10E) levels were decreased, whereas miR-874-3p levels (Fig. 10F) were increased in xenograft tumors from the sh-LINC02568 group. These findings suggested that LINC02568 downregulation increased miR-874-3p levels but decreased CCNE1 levels in breast cancer cells *in vivo*, which were in agreement with our *in vitro* study data. Therefore, LINC02568 silencing impaired breast cancer cell growth *in vivo*.

## Discussion

Several lncRNAs are aberrantly expressed in breast cancer [21,22]. Dysregulated lncRNAs function as either oncogenes or tumor suppressors to significantly contribute to breast carcinogenesis and progression [23,24]. Therefore, elucidating the roles of dysregulated lncRNAs in breast cancer may help identify novel therapeutic targets. However, how most lncRNAs are related to breast cancer remains unclear. Therefore, we investigated the specific roles and mechanisms of LINC02568 in breast cancer.

Many lncRNAs are implicated in breast cancer oncogenicity, e.g., MAFG-AS1 [25], LINC00466 [26], and LINC00649 lncRNAs [27] are overexpressed in breast cancer, exerting pro-oncogenic effects. Conversely, low LINC00982 [28], SLC16A1-AS1 [29], and EPB41L4A-AS1 lncRNA levels [30] are reported to be associated with antioncogenic functions in breast cancer. However, it is unclear how LINC02568 contributes to disease progression. Herein, LINC02568 was upregulated in breast cancer tissue and high LINC02568 levels were associated with worse overall survival. Functionally, LINC02568 depletion suppressed cell proliferation, colony formation, and metastasis, whereas overexpression exerted the opposite effects.

Many studies have reported that lncRNAs play specific roles via different mechanisms, which are closely associated with their subcellular localization [31,32]. Generally, nuclear-localized lncRNAs regulate genes via interactions with proteins [33], e.g., the lncRNA DDX11 antisense RNA 1 aggravates glioma cell malignancy by targeting HNRNPC [34]. In contrast, cytoplasmic-localized lncRNAs adsorb similar cytoplasm-enriched miRNAs, thereby suppressing their regulatory effects and indirectly modulating gene expression [35]. In our study, LINC02568 was predicted as a cytoplasm-enriched lncRNA, which was further confirmed by the nuclear–cytoplasmic fractionation experiment.

LINC02568 localization to the cytoplasm suggests that it affects breast cancer development by acting as a ceRNA. Using the starBase 3.0 and miRDB databases, miR-874-3p was predicted as a potential target of LINC02568, which was subsequently corroborated by mechanistic experiments. In addition, miR-874-3p expression was notably increased by LINC02568 knockdown but was decreased by LINC02568 upregulation in cells. Furthermore, CCNE1 was identified as a direct target of miR-874-3p in cells. Considering that LINC02568 and CCNE1 harbored the same miRNA recognition elements, we hypothesized that all three molecules may comprise a ceRNA pathway. Consequently, CCNE1 was identified as positively regulated by LINC02568, while the regulatory effect was reversed by sequestering miR-874-3p. Therefore, the LINC02568/miR-874-3p/CCNE1 regulatory network functions in breast cancer.

The expression status and functions of miR-874-3p have been characterized in different human cancer types, e.g., downregulated miR-874-3p was confirmed in hepatocellular carcinoma, which showed a notable correlation with poor differentiation, advanced staging, and prognosis [36]. miR-874-3p also had tumor suppressive roles in hepatocellular carcinoma aggressiveness [36]. In this study, miR-874-3p was successfully verified as an anticarcinogenic miRNA in breast cancer that hindered cell proliferation, colony formation, and migratory and invasive functions. Furthermore, our mechanistic investigations suggested that miR-874-3p-mediated suppressive effects in breast cancer cells were mediated by targeting CCNE1. As a member of the cyclin family, CCNE1 appears to be controlled by the LINC02568/miR-874-3p axis in breast cancer cells. Our rescue studies also showed that increased miR-874-3p or decreased CCNE1 expression reversed the LINC02568-induced increase in cell growth and motility. Therefore, LINC02568 plays a pro-oncogenic role in breast cancer progression by targeting the miR-874-3p/CCNE1 axis.

Our research had two limitations. Firstly, cell line MCF-7 was used in loss-of-function experiments, while MDA-MB-231 cells were used for gain-of-function experiments. We did not use both MCF-7 and MDA-MB-231 in all the upregulation and downregulation transfection experiments. Secondly, we did not explore the regulatory effect of LINC02568 on tumor metastasis *in vivo*. We will resolve the limitations in the near future.

In conclusion, the tumor-promoting role of LINC02568 in breast cancer cells was mediated by sequestering miR-874-3p and subsequently overexpressing CCNE1.

## Data Availability

The datasets used and/or analyzed during the current study are available from the corresponding author on reasonable request.
